# Achieving High Standards in Clinical Biochemistry: Integrating Six Sigma, Quality Goal Index (QGI), and Operating Specifications (OPSpecs) for Targeted Quality Enhancement

**DOI:** 10.7759/cureus.74693

**Published:** 2024-11-28

**Authors:** Khushbu R Panchal, Nisha D Vaghasiya, Smita H Vasava, Dharmik S Patel

**Affiliations:** 1 Department of Biochemistry, Pramukhswami Medical College, Bhaikaka University, Anand, IND; 2 Department of Biochemistry, Shantabaa Medical College, Amreli, IND

**Keywords:** laboratory quality improvement, opspec charts, quality control (qc), quality goal index (qgi), sigma metrics, total allowable error (tea)

## Abstract

Background

Sigma metrics, a cornerstone of quality control (QC) in manufacturing, have been increasingly adopted in analytical processes. In clinical biochemistry labs, Sigma analysis provides insights into the level of QC achieved and identifies deviations from perfection.

Methods

A prospective-retrospective observational study was conducted at the Central Diagnostic Laboratory of Shree Krishna Hospital between August 2021 and July 2022. Sigma metrics were calculated using the formula: Sigma = (TEa - Bias%) / CV%. Total allowable error (TEa) values were derived from guidelines including the Clinical Laboratory Improvement Amendments (CLIA), Royal College of Pathologists of Australasia (RCPA), and Rilibäk. Bias% was assessed using data from External Quality Assurance Services (EQAS), while the coefficient of variation (CV%) was calculated based on internal QC data. For parameters with Sigma values less than 3, Quality Goal Index (QGI) ratios were computed to assess whether errors were primarily due to precision or accuracy issues. Operating Specifications (OPSpecs) charts were generated to guide adjustments to internal QC procedures for underperforming tests.

Results

The analysis revealed that high-density lipoprotein (HDL) cholesterol exhibited excellent performance with a Sigma value of ≥ 6 based on newer CLIA guidelines. Parameters such as alkaline phosphatase (ALP), low-density lipoprotein (LDL) cholesterol, creatine kinase, iron, and sodium achieved Sigma values between 4 and 6, indicating satisfactory QC. However, certain parameters, including urea, creatinine, thyroid-stimulating hormone (TSH), free thyroxine (FT4), vitamin B12, and chloride, demonstrated Sigma values less than 3, signaling the need for immediate quality improvement. QGI analysis highlighted precision issues in some tests, while others showed accuracy deficits. The OPSpecs charts provided a comprehensive framework for customizing QC protocols to address specific parameter deficiencies.

Conclusions

This study highlights the value of Sigma metrics in refining QC protocols in clinical biochemistry laboratories. Shifting from generalized to parameter-specific QC plans allows for better resource allocation, improved efficiency, and higher test accuracy. Tools like QGI and OPSpecs charts help identify underperforming tests, supporting continuous quality improvement in diagnostics.

## Introduction

The importance of quality management in healthcare services, particularly clinical laboratory services, is paramount, as approximately 70% of clinical decisions are informed by laboratory results [1,2]. Quality in this context is evaluated through metrics such as accuracy, precision, sensitivity, and specificity, with quality control (QC) systems being essential for monitoring analytical processes and promptly detecting errors [3]. QC in laboratories is a dynamic, continuous process designed to ensure reliable test outcomes. Westgard's total quality management (TQM) framework involves five steps, from planning to continuous improvement, and emphasizes systematic QC strategies [3].

The laboratory testing process is divided into pre-analytical, analytical, and post-analytical phases, each contributing to potential errors, with estimates ranging from 30-75% in the pre-analytical phase, 4-30% during analysis, and 9-55% post-analysis [4]. Effective QC strategies mitigate these errors, relying on internal (IQC) and external quality control (EQC) schemes, statistical tools like Levy-Jennings charts, and the application of Westgard rules to monitor system reliability [5].

Automation and computerization in clinical laboratories have increased efficiency but also heightened the risk of false results, underscoring the ongoing need for robust QC [6]. Recent advancements incorporate Six Sigma strategies to minimize operational defects, quantifying test performance and optimizing QC practices through Sigma metrics, which denote the degree of variability and error-free results in laboratory processes [7]. Achieving a Six Sigma level reflects a defect rate of just 3.4 per million opportunities, significantly enhancing laboratory efficiency and reducing operational costs. This approach, combined with operating specifications (OPSpecs) charts, offers a clear overview of laboratory QC performance and guides optimal rejection rules based on Sigma performance [8,9].

Another important parameter for assessing laboratory performance is the quality goal index (QGI), which provides insight into whether observed errors are due to imprecision, bias, or both. A QGI value can guide laboratories in adjusting their processes to meet quality standards by highlighting specific areas requiring improvement. This enables targeted interventions to optimize laboratory accuracy and reliability.

The allowable total error (TEa), encompassing random and systematic errors, is a crucial parameter in assessing analytical performance, QC validation, and inter-laboratory comparability. Guidelines, such as those from the Clinical Laboratory Improvement Amendments (CLIA), the Guidelines ("Rili") of the German Federal Medical Council (BÄK) (Rilibäk Germany), and the Royal College of Pathologists of Australasia (RCPA), provide thresholds for acceptable error limits in biochemistry and hematology parameters [10,11,12].

Unlike most previous studies, which relied solely on CLIA standards for calculating TEa, our study will compare CLIA, Rilibäk, and RCPA standards across 37 clinical biochemistry parameters, using the results to plot OPSpecs charts, assess the QGI index, and refine laboratory QC plans.

## Materials and methods

This prospective-retrospective observational study was conducted between August 2021 and July 2022 at a NABL-accredited laboratory within a tertiary care hospital located in a rural area of Gujarat, with ethical approval granted by the institutional ethics committee (IEC/BU/2021/Ex. 39/268).

This study aimed to assess the application of the Six Sigma matrix in the clinical biochemistry laboratory, focusing on IQC, external quality assurance services (EQAS), bias, and TEa. Six Sigma calculations required TEa, bias, and coefficient of variation (CV) data. TEa values were sourced from CLIA, RCPA, and Rilibäk. Bias was derived from EQAS data. CV data was collected from IQC using equipment such as the Siemens Dimension, Advia Centaur, Gem Premier 3000, and AVL 9180 electrolyte analyzer over one year.

Internal QC material was tested daily before analyzing patient samples using Bio-Rad Control, Control L 9 Multipak, and Siemens control reagents for vitamin D. Instruments were calibrated and maintained according to NABL guidelines. Monthly EQAS materials from Bio-Rad USA and Randox USA were used to assess EQAS bias.

Internal QC followed two levels for routine clinical chemistry (normal and pathological) and three levels for immunoassay and electrolytes (Low, Normal, High), according to NABL guidelines [13]. Westgard rules (13s, 22s, R4s for rejection, and 12s for warning) were applied for result interpretation.

Calculations and data analysis

CV% was calculated for each level of control from the mean and SD of IQC data with at least 20 runs.

Equation: CV% = (SD / Mean) × 100.

Bias was calculated using the formula: Bias (%) = ((Our Mean − Peer Mean) / Peer Mean) × 100.

TEa values were obtained from CLIA, RCPA, and Rilibäk guidelines. Sigma metrics were calculated for various analytes.

Equation: Sigma (σ) = (TEa - Bias%) / CV%.

The QGI ratio was determined by: Equation: QGI = Bias / (1.5 × CV%).

TEa % (new CLIA) was taken from the Westgard online site. As of February 4, 2019, the new CLIA “proposed rule” was published in the Federal Register to expand the list of regulated analytes and define new criteria for acceptable performance for proficiency testing [10]. TEa% Rilibäk was available from the Guideline of the German Medical Association on Quality Assurance [11], and RCPA guidelines were taken from the RCPA online site [12].

In the study, sigma was calculated using three guidelines of TEa% for performance comparison. The results showed that RCPA and Rilibäk have stricter TEa% compared to CLIA. Between the new and old CLIA, the new had a narrower TEa%. It also introduced TEa% for LDL, phosphorus, amylase, and many other parameters which were not available in the previous old CLIA guideline (Table 1) [10].

**Table 1:** List of analytes with methods, instruments, and total allowable errors (Six Sigma and CLIA criteria). In the table, 'TV' refers to the target value. RANDOX EQAS (RIQAS) was used for all parameters on the Advia Centaur instrument, while Bio-Rad USA EQAS was used for all parameters on the Siemens Dimension instrument and the Gem Premier 3000. NABL: National Accreditation Board for Testing and Calibration Laboratories; CLIA: Clinical Laboratory Improvement Amendments; RCPA: Royal College of Pathologists of Australasia; RMSD: Root Mean Square Deviation; TV: Target Value; EQAS: External Quality Assurance Services; IQC: Internal Quality Control; CV: Coefficient of Variation; ALP: Alkaline Phosphatase; AST: Aspartate Aminotransferase; ALT: Alanine Aminotransferase; HDL: High-Density Lipoprotein; LDL: Low-Density Lipoprotein; CK: Creatine Kinase; LDH: Lactate Dehydrogenase; TIBC: Total Iron Binding Capacity; TSH: Thyroid-Stimulating Hormone; FT3: Free Triiodothyronine; FT4: Free Thyroxine; PSA: Prostate-Specific Antigen.

Sr. No.	Analytes (Units)	Methods	Instrument	New Criteria CLIA	Old Criteria CLIA	RCPA	Rilibak Acceptable % RMSD
1	Sodium (mmol/L)	Direct ion selective electrode	Gem premier 3000	TV ± 4 mmol/L	Same	± 12%	3.00%
2	Potassium (mmol/L)	Direct ion selective electrode	Gem premier 3000	TV ± 0.3 mmol/L	TV ± 0.5 mmol/L	± 12%	4.50%
3	Ionized Calcium (mmol/L)	Direct ion selective electrode	Gem premier 3000	TV ± 10%	Same	± 12%	7.50%
4	Chloride (mmol/L)	Direct ion selective Electrode	AVL 9180 Electrolyte Analyzer	TV ± 5%	Same	± 12%	4.50%
5	Albumin (g/dL)	Colorimetric assay with endpoint BCP method	Siemens Dimension	TV ± 8%	TV ± 10%	± 6%	12.50%
6	ALP (U/L)	Colorimetric assay IFCC method	Siemens Dimension	TV ± 20%	TV ± 30%	± 12%	11.00%
7	AST (U/L)	Colorimetric assay IFCC without Pyridoxal phosphate method	Siemens Dimension	TV ± 15%	TV ± 20%	± 12%	11.50%
8	ALT (U/L)	Colorimetric assay IFCC without Pyridoxal phosphate method	Siemens Dimension	TV ± 15%	TV ± 20%	± 12%	11.50%
9	Total Protein (g/dL)	Colorimetric Endpoint Biuret method	Siemens Dimension	TV ± 8%	TV ± 10%	± 5%	6.00%
10	Total Bilirubin (mg/dL)	Colorimetric assay with endpoint Diazo sulfanilic method	Siemens Dimension	TV ± 20%	TV ± 20% or 0.4 mg/dL	± 12%	13.00%
11	Direct Bilirubin (mg/dL)	Colorimetric assay with Jendrassik and Grof	Siemens Dimension	TV ± 20%	Same	± 12%	Not provided
12	Total Cholesterol (mg/dL)	Polychromatic endpoint	Siemens Dimension	TV ± 10%	Same	± 6%	7.00%
13	Triglycerides (mg/dL)	Enzymatic bichromatic endpoint	Siemens Dimension	TV ± 15%	TV ± 25%	± 12%	9.00%
14	HDL Cholesterol (mg/dL)	Bichromatic endpoint	Siemens Dimension	TV ± 20%	TV ± 30%	± 12%	Not provided
15	LDL Direct (mg/dL)	Colorimetric assay IFCC method	Siemens Dimension	TV ± 20%	None	± 10%	Not provided
16	Creatinine (mg/dL)	Modified Jaffe’s kinetic	Siemens Dimension	TV ± 0.2 mg/dL or ± 10%	TV ± 0.2 mg/dL or ± 15%	± 8%	11.50%
17	Urea (mg/dL)	BUN method-urease/glutamate dehydrogenase coupled enzymatic method	Siemens Dimension	TV ± 2 mg/dL or ± 9%	Same	± 12%	10.50%
18	Uric Acid (mg/dL)	Colorimetric Uricase – POD method	Siemens Dimension	TV ± 10%	TV ± 17%	± 8%	7.00%
19	Total Calcium (mg/dL)	Cresolphthalein complexone	Siemens Dimension	TV ± 1.0 mg/dL	Same	± 4%	6.00%
20	Magnesium (mg/dL)	Methyl thymol blue	Siemens Dimension	TV ± 15%	TV ± 25%	± 8%	7.50%
21	Phosphorus (mg/dL)	Phosphomolybdate UV	Siemens Dimension	TV ± 0.3 mg/dL or 10%	None	± 8%	9.00%
22	Amylase (U/L)	2-chloro-pNPG3	Siemens Dimension	TV ± 10%	TV ± 30%	± 10%	Not provided
23	Lipase (U/L)	Colorimetric	Siemens Dimension	TV ± 20%	Same	± 12%	Not provided
24	Glucose (mg/dL)	Hexokinase	Siemens Dimension	TV ± 8%	TV ± 6 mg/dL or ± 10%	± 8%	11.00%
25	CK (Creatine Kinase) (U/L)	Colorimetric assay IFCC method	Siemens Dimension	TV ± 20%	TV ± 30%	± 12%	11.00%
26	LDH (U/L)	Lactate ->Pyruvate IFCC	Siemens Dimension	TV ± 15%	TV ± 20%	± 8%	9.00%
27	Iron (mcg/dL)	Colorimetric Ferrozine method	Siemens Dimension	TV ± 15%	TV ± 20%	± 12%	Not provided
28	TIBC (Total Iron Binding Capacity) (mcg/dL)	Colorimetric FerroZine method	Siemens Dimension	TV ± 20%	None	± 8%	Not provided
29	Triiodothyronine (nmol/L)	Immunometric Assay (Competitive principle)	Advia centaur	TV ± 30%	TV ± 3SD	± 15%	15.00%
30	Thyroxin (nmol/L)	Immunometric Assay (Competitive principle)	Advia centaur	TV ± 20% or ± 1.0 mcg/dL	Same	± 15%	12.50%
31	Thyroid-Stimulating Hormone (TSH) (mIU/L)	Two-site sandwich immunoassay using direct chemiluminometric technology	Advia centaur	TV ± 20% or ± 2 mIU/L (greater)	TV ± 3SD	± 20%	13.50%
32	FT3 (Free Triiodothyronine) (pmol/L)	Direct chemiluminescent technology	Advia centaur	TV ± 0.3 ng/dL or ± 15%	TV ± 3SD	± 12%	13.00%
33	FT4 (Free Thyroxine) (pmol/L)	Direct chemiluminescent technology	Advia centaur	TV ± 0.3 ng/dL or ± 15%	TV ± 3SD	± 12%	13.00%
34	PSA Total (mcg/L)	Two-site sandwich immunoassay- direct chemiluminometric technology	Advia centaur	TV ± 0.2 ng/dL or 20%	None	± 8%	15.50%
35	Vitamin B12 (pmol/L)	Immunometric Assay (Competitive principle)	Advia centaur	TV ± 25%	TV ± 30%	± 15%	Not provided
36	Vitamin D3 (nmol/L)	Immunometric Assay (Competitive principle)	Advia centaur	TV ± 20%	None	± 15%	Not provided
37	Ferritin (mcg/L)	Immunometric Assay (sandwich principle)	Advia centaur	TV ± 25%	Same	± 10%	Not provided

Normalized OPSpecs charts were created to establish the QC guidelines for various parameters. The first step was to select the appropriate chart based on two factors: a) The number of controls run each day (N=2 or N=3) b) The level of analytical quality assurance (90% or 50%).

We selected the N=2, 50% AQA chart, as two levels of controls are run twice daily. This approach allows for error detection in the second run if missed in the first. The X and Y axis values were calculated using the normalized OPSpecs calculator available on the Westgard website [14]. Pre-made charts were also downloaded from the website, and the final values were plotted and transferred to a PowerPoint slide for presentation.

The study included all values obtained from IQC and EQAS samples. Parameters were excluded from the analysis if there were no available TEa values in any guidelines, if the parameters were not part of the EQAS programs, or if the parameters were rare and lacked sufficient IQC data to calculate their CV.

Statistical analysis

Data were processed using Microsoft Excel for mean, SD, CV, bias, and sigma calculations. The normalized OPSpecs chart calculator from Westgard was used to plot OPSpecs charts.

## Results

The analysis of IQC data showed varying performance across clinical chemistry, electrolytes, and immunoassay parameters. CV% for each analyte were calculated for different levels (L1, L2, L3). Most analytes performed within the established thresholds, though some displayed higher bias, indicating areas for improvement in analytical precision and accuracy. Bias percentages were assessed by comparing the laboratory's results with peer data from EQAS, and TEa values, derived from CLIA, RCPA, and Rilibäk guidelines, were used to evaluate overall analytical performance.

As mentioned in Table 2, the maximum CV% among the three QCs was calculated into the sigma matrix.

**Table 2:** CV% and bias% for clinical chemistry, immunoassay, and electrolyte parameters. ALT: Alanine Aminotransferase; ALP: Alkaline Phosphatase; AST: Aspartate Aminotransferase; HDL: High-Density Lipoprotein; LDL: Low-Density Lipoprotein; TIBC: Total Iron Binding Capacity; T3: Triiodothyronine; T4: Thyroxine; TSH: Thyroid-Stimulating Hormone; FT4: Free Thyroxine; FT3: Free Triiodothyronine; PSA: Prostate-Specific Antigen; LDH: Lactate Dehydrogenase; CV%: Coefficient of Variation Percent.

S. No.	Analyte	Bias%	CV%
1	ALT	1.29	4.79
2	Albumin	2.72	2.3
3	ALP	3.05	3.03
4	AST	1.76	4.64
5	Bilirubin, total	2.89	5.65
6	Bilirubin Direct	3.92	7.45
7	Total Protein	2.09	2.05
8	Cholesterol total	2.66	2.27
9	Cholesterol (HDL)	3.11	2.95
10	Cholesterol (LDL)	5.05	3.59
11	Triglycerides	4.87	2.79
12	Urea	2.98	4.21
13	Creatinine	4.12	2.97
14	Uric Acid	2.49	2.21
15	Calcium, total	3.42	2.89
16	Magnesium	1.89	4.16
17	Phosphorus	1.9	2.5
18	Amylase	2.36	2.05
19	Lipase	8.8	3.46
20	Glucose	3	1.7
21	Creatine kinase	3.65	3.14
22	LDH	2.05	4.63
23	Iron	3.04	3.19
24	TIBC	4.97	4.48
25	T3	4.47	7.51
26	T4	2.58	6.64
27	TSH	3.39	8.33
28	FT4	3.49	7.35
29	FT3	3.16	4
30	Total PSA	5.48	5.15
31	Vitamin B12	5.66	9.42
32	Vitamin D	5.08	8.19
33	Ferritin	2.24	5.72
34	Sodium	0.98	0.77
35	Potassium	1.75	1.98
36	Chloride	1.34	3.29
37	Ionized calcium	2.72	1.99

As shown in Table 3, the Sigma values for clinical chemistry, immunoassay, and electrolyte parameters were evaluated using three different guidelines.

**Table 3:** Sigma values by three different guidelines for clinical chemistry, immunoassay, and electrolyte parameters. ALT: Alanine Aminotransferase; ALP: Alkaline Phosphatase; AST: Aspartate Aminotransferase; HDL: High-Density Lipoprotein; LDL: Low-Density Lipoprotein; TIBC: Total Iron Binding Capacity; T3: Triiodothyronine; T4: Thyroxine; TSH: Thyroid-Stimulating Hormone; FT4: Free Thyroxine; FT3: Free Triiodothyronine; PSA: Prostate-Specific Antigen; LDH: Lactate Dehydrogenase; CLIA: Clinical Laboratory Improvement Amendments; RCPA: Royal College of Pathologists of Australasia; NA: Not Available; NC: Not Calculated.

Sr. No.	Analytes	Sigma (New CLIA)	Sigma (Old CLIA)	Sigma (RCPA)	Sigma (Rilibak)
1	ALT	3.13	4.25	2.46	2.35
2	Albumin	3.07	4.04	2.1	5.25
3	ALP	5.81	9.28	3.04	2.69
4	AST	3.12	4.26	2.43	2.32
5	Bilirubin, total	3.11	3.11	1.65	1.83
6	Bilirubin, direct	NA	NA	2.31	NA
7	Total Protein	3.19	4.28	1.56	2.1
8	Cholesterol, total	3.43	3.43	1.56	2.02
9	Cholesterol (HDL)	6.09	9.67	3.23	NA
10	Cholesterol (LDL)	5.4	8.88	1.91	NA
11	Triglycerides	3.84	7.58	2.72	1.6
12	Urea	1.82	1.82	2.68	2.25
13	Creatinine	1.98	3.74	1.28	2.51
14	Uric Acid	3.53	6.9	2.57	2.08
15	Calcium, total	3.16	3.16	0.58	1.44
16	Magnesium	3.33	5.84	1.57	1.45
17	Phosphorus	3.3	NA	2.47	2.88
18	Amylase	3.91	14.1	3.91	NA
19	Lipase	NA	NA	4.07	NA
20	Glucose	3.24	1.99	3.24	5.12
21	Creatine kinase	5.3	8.52	2.72	2.4
22	LDH	3.12	4.3	1.45	1.69
23	Iron	4.11	5.8	3.09	NA
24	TIBC	3.51	NA	0.81	NA
25	T3	3.44	NC	1.42	1.42
26	T4	3.12	3.12	2.25	1.82
27	TSH	2.14	NC	2.14	1.35
28	FT4	1.65	NC	1.22	1.36
29	FT3	NA	NA	4.35	2.54
30	Total PSA	3.13	NA	0.65	2.2
31	Vitamin B12	2.2	2.74	1.14	NA
32	Vitamin D	NA	NA	1.36	NA
33	Ferritin	3.21	NA	2.33	2.07
34	Sodium	4.05	4.05	1.39	2.72
35	Potassium	3.65	4.81	1.92	1.63
36	Chloride	1.2	1.2	0.54	1.03
37	Ionized calcium	NA	NA	NA	3.11

New CLIA vs. old CLIA

Parameters such as low-density lipoprotein (LDL), alkaline phosphatase (ALP), triglycerides, uric acid, amylase, and creatine kinase, previously classified as ≥ 6 Sigma under old CLIA, now fall into the 4-6 or 3-4 categories under new CLIA. HDL remains consistent in the ≥ 6 Sigma category across both guidelines, indicating stricter TEa% in the new CLIA (Table 4).

**Table 4:** Consolidated sigma classification across guidelines. CLIA: Clinical Laboratory Improvement Amendments; RCPA: Royal College of Pathologists of Australasia; HDL: High-Density Lipoprotein; LDL: Low-Density Lipoprotein; ALP: Alkaline Phosphatase; ALT: Alanine Aminotransferase; AST: Aspartate Aminotransferase; TIBC: Total Iron Binding Capacity; TP: Total Protein; LDH: Lactate Dehydrogenase; FT3: Free Triiodothyronine; T3: Triiodothyronine; T4: Thyroxine; PSA: Prostate-Specific Antigen; FT4: Free Thyroxine; TSH: Thyroid-Stimulating Hormone.

Sigma Category	New CLIA	Old CLIA	RCPA	Rilibak
≥6 (World Class)	HDL	HDL, LDL, ALP, Triglycerides, Uric Acid, Amylase, Creatine Kinase	None	None
4-6 (Excellent)	ALP, LDL, Creatine Kinase, Iron, Sodium	ALT, AST, Albumin, Total Protein, Magnesium, LDH, Iron, Sodium, Potassium	Lipase, FT3	Albumin, Glucose
3-4 (Good/Marginal)	ALT, Albumin, AST, TIBC, TP, Cholesterol Total, Triglyceride, Uric Acid, Calcium Total, Magnesium, Phosphorus, Amylase, Glucose, LDH, TIBC, T3, T4, Total PSA, Ferritin, Potassium	Bilirubin Total, Cholesterol Total, Creatinine, Calcium Total, Total T4	ALP, HDL, Amylase, Glucose, Iron	Ionized Calcium
<3 (Poor)	Urea, Creatinine, TSH, FT4, Vitamin B12, Chloride	Urea, Glucose, Vitamin B12, Chloride	Most parameters	Most parameters

New CLIA vs. RCPA

Under RCPA, no parameters achieve ≥ 6 Sigma, and HDL is downgraded to the 3-4 category. This reflects RCPA’s stricter TEa% compared to New CLIA (Table 4).

New CLIA vs. Rilibäk

Rilibäk places no parameters in the ≥ 6 Sigma category. Albumin and Glucose, classified as 3-4 under New CLIA, are upgraded to 4-6 under Rilibäk. However, Rilibäk has more parameters in the < 3 category, highlighting its stricter TEa% (Table 4).

RCPA vs. Rilibäk

Rilibäk is less stringent for some parameters, placing Albumin and Glucose in the 4-6 category, while RCPA classifies them as < 3 and 3-4, respectively. Both guidelines have a high proportion of parameters in the < 3 category, but Rilibäk demonstrates stricter thresholds overall (Table 4).

Quality goal index (QGI)

The Quality Goal Index (QGI) was calculated to identify whether quality issues were due to imprecision (IQC/CV%) or inaccuracy (EQAS/Bias%) (Table 5).

**Table 5:** Quality goal index (QGI). QGI = Bias/1.5 × CV%. Bias%: Percentage Bias; CV%: Coefficient of Variation Percent; FT4: Free Thyroxine; TSH: Thyroid-Stimulating Hormone.

Analyte	Bias%	CV%	QGI	Interpretation
Bilirubin, direct	3.92	7.45	0.35	Imprecision
Creatinine	4.12	2.97	0.92	Imprecision and Inaccuracy
Urea	2.98	4.21	0.47	Imprecision
Chloride	1.34	3.16	0.28	Imprecision
FT4	3.49	7.35	0.32	Imprecision
TSH	3.39	8.33	0.27	Imprecision
Vitamin D	5.08	8.19	0.41	Imprecision
Vitamin B12	5.66	9.42	0.40	Imprecision

QGI < 0.8: Imprecision

QGI > 1.2: Inaccuracy

QGI 0.8-1.2: Both imprecision and inaccuracy

New CLIA guidelines were used, along with Rilibäk and RCPA guidelines for parameters lacking TEa%.

Out of 9 parameters, 8 were imprecise, indicating issues with IQC, while creatinine showed errors in both IQC and EQAS.

Normalized OPSpecs charts

OPSpecs charts were generated to determine QC guidelines based on the lab's sigma levels, using observed CV% and EQAS controls as bias indicators. Two QC levels were run across all parameters, with an N=2 and a 50% AQA OPSpecs chart applied for the analysis.

**Figure 1:**
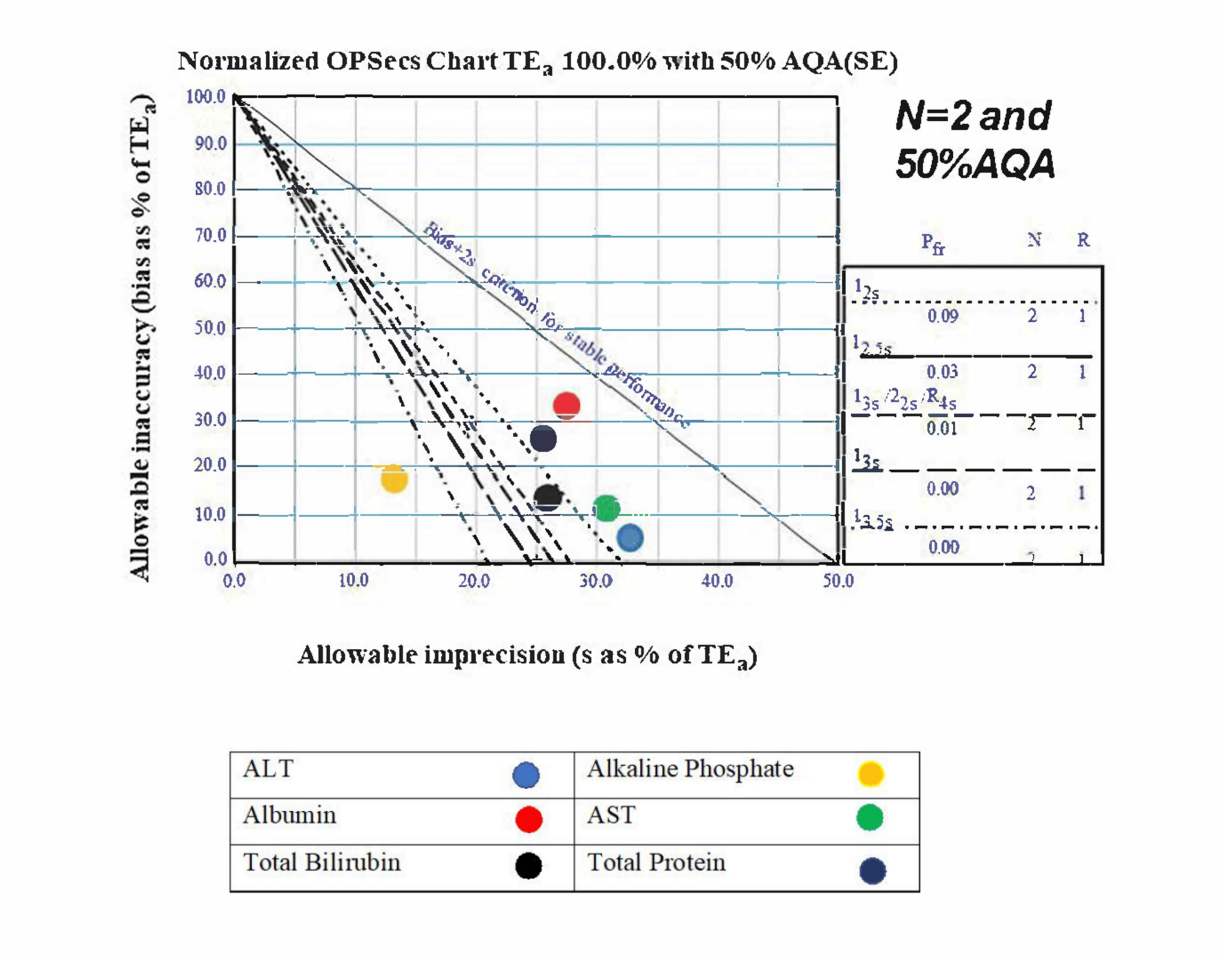
OPSpecs chart for liver function test parameters with sigma level, QC analysis, and 50% AQA threshold (N=2). This figure presents a normalized OPSpecs chart for TEₐ set at 100.0%, with a 50% AQA threshold. The chart displays the relationship between allowable inaccuracy (bias) as a percentage of TEₐ on the y-axis and allowable imprecision (standard deviation, s) as a percentage of TEₐ on the x-axis. Biochemical parameters represented include ALT (blue circle), Alkaline Phosphatase (yellow circle), Albumin (red circle), AST (green circle), Total Bilirubin (black circle), and Total Protein (dark blue circle). The diagonal dashed lines depict performance criteria for stable measurements based on allowable bias and imprecision limits. The inset details QC rules such as 1₂₅s and 2₁₂.₅s, which are statistical thresholds applied to ensure consistent measurement accuracy in clinical laboratories. Here, 'N = 2' denotes two measurement replicates, 'Pff' reflects the chance of a test falsely failing, and 'R' indicates the number of repeated measures considered. OPSpecs: Operating Specifications; TEₐ: Total Allowable Error; AQA: Analytical Quality Assurance; QC: Quality Control; ALT: Alanine Aminotransferase; AST: Aspartate Aminotransferase; Pff: Probability of False Failure; R: Replication.

**Figure 2:**
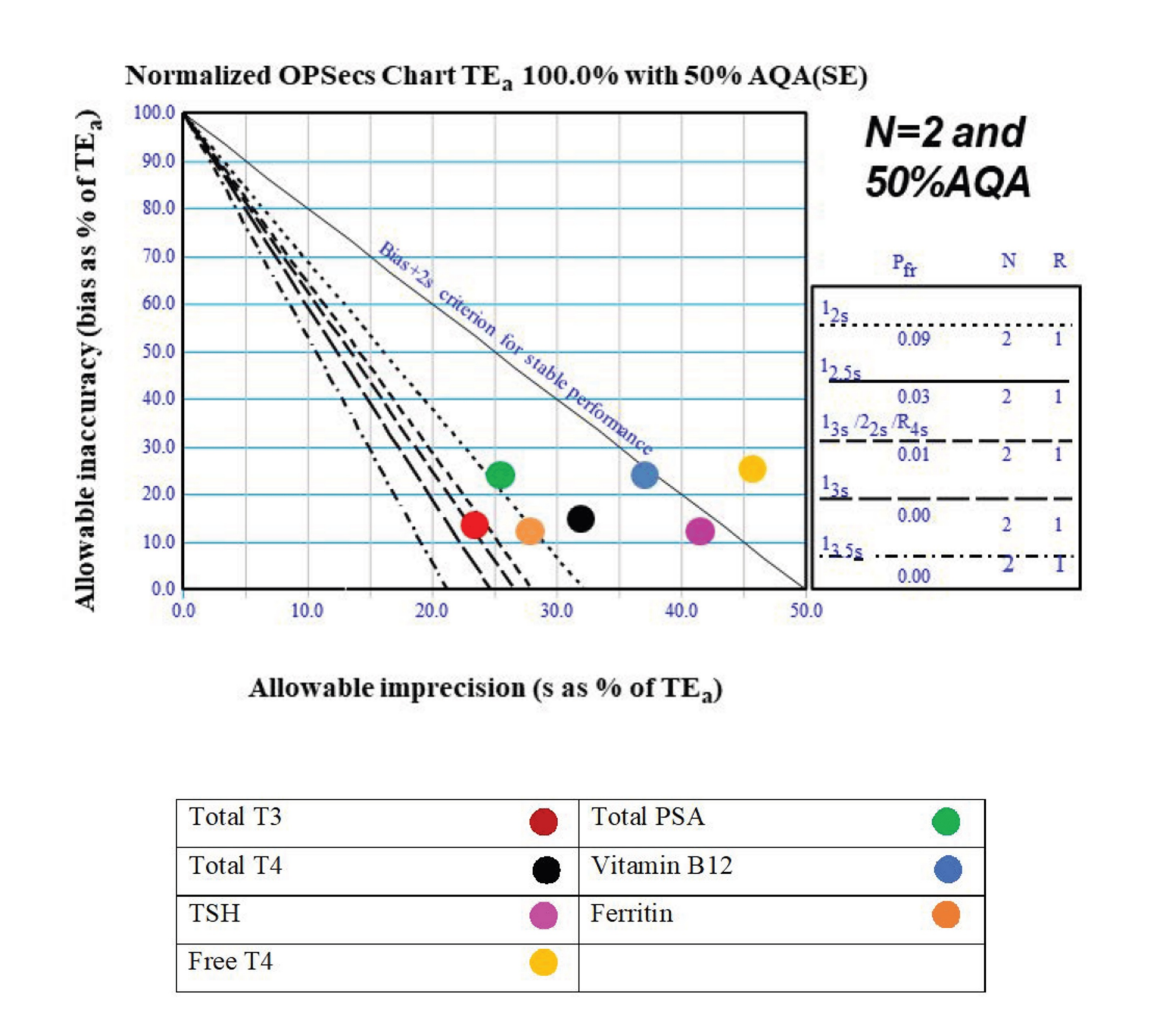
OPSpecs chart for immunoassay parameters with sigma level, QC analysis, and 50% AQA threshold (N=2). This figure presents a normalized OPSpecs chart for total allowable error (TEₐ) set at 100.0% with a 50% analytical quality assurance (AQA) threshold. The chart illustrates the relationship between allowable inaccuracy (bias) as a percentage of TEₐ (y-axis) and allowable imprecision (standard deviation, s) as a percentage of TEₐ (x-axis). The biochemical parameters represented include total T3 (triiodothyronine, red circle), total T4 (thyroxine, black circle), TSH (thyroid-stimulating hormone, magenta circle), free T4 (yellow circle), total PSA (prostate-specific antigen, green circle), vitamin B12 (cyan circle), and ferritin (orange circle). The diagonal dashed lines represent performance criteria for stable measurements based on allowable bias and imprecision limits. The inset details quality control (QC) rules such as 1₂₅s and 2₁₂.₅s, which are statistical thresholds used to maintain precision and accuracy in clinical laboratory testing. Here, 'N = 2' indicates two measurement replicates, 'Pff' (probability of false failure) denotes the likelihood of a test falsely failing based on QC criteria, and 'R' (replication) represents the number of repeated measures for evaluation.

We analyzed and generated OPSpecs charts for all 37 parameters to establish QC guidelines based on the lab’s achieved sigma levels, incorporating observed CV% and EQAS controls as bias indicators. Although OPSpecs charts were created for each parameter, we present only two representative charts here for conciseness (Figures 1-2).

## Discussion

This study analyzed data from daily IQC and EQAS over one year, adhering to NABL accreditation standards. Bias, indicating method accuracy, was assessed via EQAS, and sigma metrics were calculated, with values ranging from 0 to 6, higher values represent better performance. A sigma level of 3 is minimally acceptable; below 3 indicates instability. Of the 37 tested parameters, 9 had sigma levels below 3, and 27 exceeded this threshold. The QGI ratio was used to identify issues in imprecision (IQC CV%) or inaccuracy (EQAS Bias%), with QGI values for the 9 parameters below 3 presented in Table 5.

Previous studies provide a comparative context for the present findings. For example, Bhavna S et al. reported high sigma values (>6) for triglycerides, creatine kinase (CK), and amylase using an Olympus AU 400 analyzer. In our study, parameters such as HDL and ALP also achieved >6 sigma values, in line with new CLIA guidelines, while our results for urea, sodium, and potassium showed better sigma metrics than in their study [15].

Similarly, Kumar BV et al. evaluated 16 parameters over one year at a secondary care hospital, reporting sigma values ≥6 for four analytes (ALP, magnesium, triglyceride, HDL) and poor performance (sigma <3) for others, including urea and potassium. Our findings for urea and potassium showed improved sigma levels under updated CLIA standards, and QGI for urea was consistent, indicating imprecision [16].

Ramteke TD et al. examined data from two automated analyzers at a tertiary care hospital and found excellent sigma metrics for HDL, LDL, ALP, triglycerides, and CK. Our study achieved similar performance levels for HDL, LDL, and ALP, while urea and chloride exhibited sigma values below 3, indicating a need for quality improvement [17]. Nanda S et al. reported sigma values above 6 for parameters including ALP and uric acid, findings consistent with ours. In our study, triglycerides achieved sigma levels >6, aligning with their results [8].

Gami B et al. analyzed IQC and EQAS data for sodium and potassium over nine months, finding significant variability in sigma metrics based on the TEa guidelines used. They observed that electrolytes with narrow biological variation often have lower sigma values due to stricter TEa limits, with factors like upgraded equipment and methodological differences also influencing results. Similarly, our study found sigma values below 3 for sodium and potassium in some cases, even with updated CLIA standards. These findings align with Westgard S et al.’s conclusions, emphasizing the need for appropriate TEa guidelines and considering both analytical and biological factors when evaluating sigma metrics for laboratory quality improvement [18,19].

Iqbal S and Mustansar T noted acceptable sigma metrics (≥3) for glucose, cholesterol, and HDL, but low values for chloride, albumin, and total bilirubin due to stricter TEa limits, similar to our findings for sodium and potassium. Maksane S et al. reported sigma >6 for ALP, AST, and triglycerides, aligning with our high HDL and ALP performance. While their urea results ranged from 3-6 sigma, ours showed better metrics under updated CLIA standards, reflecting improved precision and bias. Both studies highlight the need for tailored quality control to address analytes with low sigma metrics and enhance overall laboratory quality [20,21].

The comparison highlights similarities and differences in sigma metrics between your study and Panda CR et al.’s findings. Both identified uric acid, total cholesterol, and iron as world-class performers, with glucose, total protein, and triglycerides showing good reliability. Key differences include HDL, which was "unacceptable" in Panda CR et al.’s study but world-class in yours, and potassium, which performed better in your study. Creatinine and chloride were consistently poor across both studies. Your broader evaluation across multiple quality systems revealed variations likely due to differences in calibration, equipment, or procedural rigor, underscoring the importance of robust quality control [22].

The QC rejection rules in this study are based on sigma performance. For 6 Sigma tests, the 13s rule is sufficient, requiring two measurements per run (N=2, R=1). At 5 Sigma, the 13s/22s/R4s rule set is used, also with two measurements per run (N=2, R=1). For 4 Sigma, four rules (13s/22s/R4s/41s) are needed, with either four measurements per run (N=4, R=1) or two measurements across two runs (N=2, R=2), using the 41s rule for monitoring. Below 4 Sigma, a multi-rule procedure, including the 8x rule, is applied with four measurements across two runs (N=4, R=2) or two measurements over four runs (N=2, R=4).

The Six Sigma methodology effectively assesses analytical quality and optimizes QC rules based on sigma values, ensuring tailored IQC practices for each test. Sigma metrics allow integrated evaluation of both IQC and EQAS schemes, enhancing laboratory quality. The OPSpecs chart enables laboratories to customize QC procedures, improving performance for specific test needs [8,9].

The study found parameters that indicated areas for quality improvement with sigma levels less than 3. For instance, the QGI verified that potassium and urea performed poorly because of inaccuracy (high EQAS Bias%) and imprecision (high IQC CV%). Similar difficulties with stringent TEa limits and limited biological variation were encountered by sodium and chloride, necessitating exact analytical techniques. Better calibration, updated machinery, and improved procedures to increase accuracy and stability may all be part of improving these areas.

Sigma metrics can be standardized and customized to scale across labs. Standardization entails bringing procedures into compliance with new TEa regulations, such as CLIA and routine equipment calibration. In environments with limited resources, customization enables labs to concentrate on high-impact analytes for Sigma-based QC and progressively expand the methodology. A flexible and efficient quality system is made possible by tools such as OPSpecs chart, which support customizing QC strategies for particular tests.

This study uniquely integrates IQC and EQAS to evaluate 37 parameters, identifying both strengths (e.g., HDL, ALP) and weaknesses (e.g., sodium, potassium). Unlike other studies, it compares performance under both new and old CLIA standards, as well as alternative guidelines like Rilibak and RCPA, providing a broader, global perspective. Tailored QC strategies, such as using OPSpecs charts based on sigma metrics, make its recommendations practical and adaptable to diverse settings. By addressing challenges like stricter TEa limits and offering solutions such as recalibration and procedural improvements, the study contributes significantly to advancing laboratory quality practices.

Limitations

it was conducted in a single laboratory, which may affect the generalizability of the findings. The use of retrospective data could introduce biases, and the exclusion of other clinically significant parameters limits the results. Bias assessment and sigma metrics relied on standardized EQAS and IQC data, which may vary. Additionally, the study did not evaluate the feasibility of implementing proposed quality control rules in different laboratories or their impact on clinical outcomes, suggesting the need for further multi-center research.

## Conclusions

This study highlights the value of sigma metrics in advancing quality standards in clinical biochemistry laboratories while emphasizing their potential impact on patient care. By combining IQC and EQAS data, sigma metrics offer a robust framework for evaluating laboratory performance and identifying areas for improvement. Parameter-specific quality control adjustments, guided by tools such as QGI ratios and OPSpecs charts, enhance test accuracy and reliability. These improvements not only streamline laboratory workflows but also reduce the likelihood of errors that could compromise clinical decision-making. Regular incorporation of sigma metrics into practice ensures ongoing quality improvements, fostering greater diagnostic precision, timely interventions, and better patient outcomes.
